# Analysing Complex Problem-Solving Strategies from a Cognitive Perspective: The Role of Thinking Skills

**DOI:** 10.3390/jintelligence10030046

**Published:** 2022-07-25

**Authors:** Hao Wu, Gyöngyvér Molnár

**Affiliations:** 1 MTA-SZTE Digital Learning Technologies Research Group, Center for Learning and Instruction, University of Szeged, 6722 Szeged, Hungary; 2 MTA-SZTE Digital Learning Technologies Research Group, Institute of Education, University of Szeged, 6722 Szeged, Hungary; gymolnar@edpsy.u-szeged.hu

**Keywords:** complex problem solving, thinking skills, logfile analysis, process data

## Abstract

Complex problem solving (CPS) is considered to be one of the most important skills for successful learning. In an effort to explore the nature of CPS, this study aims to investigate the role of inductive reasoning (IR) and combinatorial reasoning (CR) in the problem-solving process of students using statistically distinguishable exploration strategies in the CPS environment. The sample was drawn from a group of university students (N = 1343). The tests were delivered via the eDia online assessment platform. Latent class analyses were employed to seek students whose problem-solving strategies showed similar patterns. Four qualitatively different class profiles were identified: (1) 84.3% of the students were proficient strategy users, (2) 6.2% were rapid learners, (3) 3.1% were non-persistent explorers, and (4) 6.5% were non-performing explorers. Better exploration strategy users showed greater development in thinking skills, and the roles of IR and CR in the CPS process were varied for each type of strategy user. To sum up, the analysis identified students’ problem-solving behaviours in respect of exploration strategy in the CPS environment and detected a number of remarkable differences in terms of the use of thinking skills between students with different exploration strategies.

## 1. Introduction

Problem solving is part and parcel of our daily activities, for instance, in determining what to wear in the morning, how to use our new electronic devices, how to reach a restaurant by public transport, how to arrange our schedule to achieve the greatest work efficiency and how to communicate with people in a foreign country. In most cases, it is essential to solve the problems that recur in our study, work and daily lives. These situations require problem solving. Generally, problem solving is the thinking that occurs if we want “to overcome barriers between a given state and a desired goal state by means of behavioural and/or cognitive, multistep activities” (Frensch and Funke 1995, p. 18). It has also been considered as one of the most important skills for successful learning in the 21st century. This study focuses on one specific kind of problem solving, complex problem solving (CPS). (Numerous other terms are also used (Funke et al. 2018), such as interactive problem solving (Greiff et al. 2013; Wu and Molnár 2018), and creative problem solving (OECD 2010), etc.).

CPS is a transversal skill (Greiff et al. 2014), operating several mental activities and thinking skills (see Molnár et al. 2013). In order to explore the nature of CPS, some studies have focused on detecting its component skills (Wu and Molnár 2018), whereas others have analysed students’ behaviour during the problem-solving process (Greiff et al. 2018; Wu and Molnár 2021). This study aims to link these two fields by investigating the role of thinking skills in learning by examining students’ use of statistically distinguishable exploration strategies in the CPS environment.

### 1.1. Complex Problem Solving: Definition, Assessment and Relations to Intelligence

According to a widely accepted definition proposed by Buchner (1995), CPS is “the successful interaction with task environments that are dynamic (i.e., change as a function of users’ intervention and/or as a function of time) and in which some, if not all, of the environment’s regularities can only be revealed by successful exploration and integration of the information gained in that process” (Buchner 1995, p. 14). A CPS process is split into two phases, knowledge acquisition and knowledge application. In the knowledge acquisition (KAC) phase of CPS, the problem solver understands the problem itself and stores the acquired information (Funke 2001; Novick and Bassok 2005). In the knowledge application (KAP) phase, the problem solver applies the acquired knowledge to bring about the transition from a given state to a goal state (Novick and Bassok 2005).

Problem solving, especially CPS, has frequently been compared or linked to intelligence in previous studies (e.g., Beckmann and Guthke 1995; Stadler et al. 2015; Wenke et al. 2005). Lotz et al. (2017) observed that “intelligence and [CPS] are two strongly overlapping constructs” (p. 98). There are many similarities and commonalities that can be detected between CPS and intelligence. For instance, CPS and intelligence share some of the same key features, such as the integration of information (Stadler et al. 2015). Furthermore, Wenke et al. (2005) stated that “the ability to solve problems has featured prominently in virtually every definition of human intelligence” (p. 9); meanwhile, from the opposite perspective, intelligence has also been considered as one of the most important predictors of the ability to solve problems (Wenke et al. 2005). Moreover, the relation between CPS and intelligence has also been discussed from an empirical perspective. A meta-analysis conducted by Stadler et al. (2015) selected 47 empirical studies (total sample size N = 13,740) which focused on the correlation between CPS and intelligence. The results of their analysis confirmed that a correlation between CPS and intelligence exists with a moderate effect size of M(g) = 0.43.

Due to the strong link between CPS and intelligence, assessments of these two domains have been connected and have overlapped to a certain extent. For instance, Beckmann and Guthke (1995) observed that some of the intelligence tests “capture something akin to an individual’s general ability to solve problems (e.g., Sternberg 1982)” (p. 184). Nowadays, some widely used CPS assessment methods are related to intelligence but still constitute a distinct construct (Schweizer et al. 2013), such as the MicroDYN approach (Greiff and Funke 2009; Greiff et al. 2012; Schweizer et al. 2013). This approach uses the minimal complex system to simulate simplistic, artificial but still complex problems following certain construction rules (Greiff and Funke 2009; Greiff et al. 2012).

The MicroDYN approach has been widely employed to measure problem solving in a well-defined problem context (i.e., “problems have a clear set of means for reaching a precisely described goal state”, Dörner and Funke 2017, p. 1). To complete a task based on the MicroDYN approach, the problem solver engages in dynamic interaction with the task to acquire relevant knowledge. It is not possible to create this kind of test environment with the traditional paper-and-pencil-based method. Therefore, it is currently only possible to conduct a MicroDYN-based CPS assessment within the computer-based assessment framework. In the context of computer-based assessment, the problem-solvers’ operations were recorded and logged by the assessment platform. Thus, except for regular achievement-focused result data, logfile data are also available for analysis. This provides the option of exploring and monitoring problem solvers’ behaviour and thinking processes, specifically, their exploration strategies, during the problem-solving process (see, e.g., Chen et al. 2019; Greiff et al. 2015a; Molnár and Csapó 2018; Molnár et al. 2022; Wu and Molnár 2021).

Problem solving, in the context of an ill-defined problem (i.e., “problems have no clear problem definition, their goal state is not defined clearly, and the means of moving towards the (diffusely described) goal state are not clear”, Dörner and Funke 2017, p. 1), involved a different cognitive process than that in the context of a well-defined problem (Funke 2010; Schraw et al. 1995), and it cannot be measured with the MicroDYN approach. The nature of ill-defined problem solving has been explored and discussed in numerous studies (e.g., Dörner and Funke 2017; Hołda et al. 2020; Schraw et al. 1995; Welter et al. 2017). This will not be discussed here as this study focuses on well-defined problem solving.

### 1.2. Inductive and Combinatorial Reasoning as Component Skills of Complex Problem Solving

Frensch and Funke (1995) constructed a theoretical framework that summarizes the basic components of CPS and the interrelations among the components. The framework contains three separate components: problem solver, task and environment. The impact of the problem solver is mainly relevant to three main categories, which are memory contents, dynamic information processing and non-cognitive variables. Some thinking skills have been reported to play an important role in dynamic information processing. We can thus describe them as component skills of CPS. Inductive reasoning (IR) and combinatorial reasoning (CR) are the two thinking skills that have been most frequently discussed as component skills of CPS.

IR is the reasoning skill that has been covered most commonly in the literature. Currently, there is no universally accepted definition. Molnár et al. (2013) described it as the cognitive process of acquiring general regularities by generalizing single and specific observations and experiences, whereas Klauer (1990) defined it as the discovery of regularities that relies upon the detection of similarities and/or dissimilarities as concerns attributes of or relations to or between objects. Sandberg and McCullough (2010) provided a general conclusion of the definitions of IR: it is the process of moving from the specific to the general.

Csapó (1997) pointed out that IR is a basic component of thinking and that it forms a central aspect of intellectual functioning. Some studies have also discussed the role of IR in a problem-solving environment. For instance, Mayer (1998) stated that IR will be applied in information processing during the process of solving general problems. Gilhooly (1982) also pointed out that IR plays a key role in some activities in the problem-solving process, such as hypothesis generation and hypothesis testing. Moreover, the influence of IR on both KAC and KAP has been analysed and demonstrated in previous studies (Molnár et al. 2013).

Empirical studies have also provided evidence that IR and CPS are related. Based on the results of a large-scale assessment (N = 2769), Molnár et al. (2013) showed that IR significantly correlated with 9–17-year-old students’ domain-general problem-solving achievement (r = 0.44–0.52). Greiff et al. (2015b) conducted a large-scale assessment project (N = 2021) in Finland to explore the links between fluid reasoning skills and domain-general CPS. The study measured fluid reasoning as a two-dimensional model which consisted of deductive reasoning and scientific reasoning and included inductive thinking processes (Greiff et al. 2015b). The results drawing on structural equation modelling indicated that fluid reasoning which was partly based on IR had significant and strong predictive effects on both KAC (β = 0.51) and KAP (β = 0.55), the two phases of problem solving. Such studies have suggested that IR is one of the component skills of CPS.

According to Adey and Csapó’s (2012) definition, CR is the process of creating complex constructions out of a set of given elements that satisfy the conditions explicitly given in or inferred from the situation. In this process, some cognitive operations, such as combinations, arrangements, permutations, notations and formulae, will be employed (English 2005). CR is one of the basic components of formal thinking (Batanero et al. 1997). The relationship between CR and CPS has frequently been discussed. English (2005) demonstrated that CR has an essential meaning in several types of problem situations, such as problems requiring the systematic testing of alternative solutions. Moreover, Newell (1993) pointed out that CR is applied in some key activities of problem-solving information processing, such as strategy generation and application. Its functions include, but are not limited to, helping problem solvers to discover relationships between certain elements and concepts, promoting their fluency of thinking when they are considering different strategies (Csapó 1999) and identifying all possible alternatives (OECD 2014). Moreover, Wu and Molnár’s (2018) empirical study drew on a sample (N = 187) of 11–13-year-old primary school students in China. Their study built a structural equation model between CPS, IR and CR, and the result indicated that CR showed a strong and statistically significant predictive power for CPS (β = 0.55). Thus, the results of the empirical study also support the argument that CR is one of the component skills of CPS.

### 1.3. Behaviours and Strategies in a Complex Problem-Solving Environment

Wüstenberg et al. (2012) stated that the creation and implementation of strategic exploration are core actions of the problem-solving task. Exploring and generating effective information are key to successfully solving a problem. Wittmann and Hattrup (2004) illustrated that “riskier strategies [create] a learning environment with greater opportunities to discover and master the rules and boundaries [of a problem]” (p. 406). Thus, when gathering information about a complex problem, there may be differences between exploration strategies in terms of efficacy. The MicroDYN scenarios, a simplification and simulation of the real-world problem-solving context, will also be influenced by the adoption and implementation of exploration strategies.

The effectiveness of the isolated variation strategy (or “Vary-One-Thing-At-A-Time” strategy—VOTAT; Vollmeyer et al. 1996) in a CPS environment has been hotly debated (Chen et al. 2019; Greiff et al. 2018; Molnár and Csapó 2018; Molnár et al. 2022; Wu and Molnár 2021; Wüstenberg et al. 2014). To use the VOTAT strategy, a problem solver “systematically varies only one input variable, whereas the others remain unchanged. This way, the effect of the variable that has just been changed can be observed directly by monitoring the changes in the output variables” (Molnár and Csapó 2018, p. 2). Understanding and using VOTAT effectively is the foundation for developing more complex strategies for coordinating multiple variables and the basis for some phases of scientific thinking (i.e., inquiry, analysis, inference and argument; Kuhn 2010; Kuhn et al. 1995).

Some previous studies have indicated that students who are able to apply VOTAT are more likely to achieve higher performance in a CPS assessment (Greiff et al. 2018), especially if the problem is a well-defined minimal complex system (such as MicroDYN) (Fischer et al. 2012; Molnár and Csapó 2018; Wu and Molnár 2021). For instance, Molnár and Csapó (2018) conducted an empirical study to explore how students’ exploration strategies influence their performance in an interactive problem-solving environment. They measured a group (N = 4371) of 3rd- to 12th-grade (aged 9–18) Hungarian students’ problem-solving achievement and modelled students’ exploration strategies. This result confirmed that students’ exploration strategies influence their problem-solving performance. For example, conscious VOTAT strategy users proved to be the best problem-solvers. Furthermore, other empirical studies (e.g., Molnár et al. 2022; Wu and Molnár 2021) achieved similar results, thus confirming the importance of VOTAT in a MicroDYN-based CPS environment.

Lotz et al. (2017) illustrated that effective use of VOTAT is associated with higher levels of intelligence. Their study also pointed out that intelligence has the potential to facilitate successful exploration behaviour. Reasoning skills are an important component of general intelligence. Based on Lotz et al.’s (2017) statements, the roles IR and CR play in the CPS process might vary due to students’ different strategy usage patterns. However, there is still a lack of empirical studies in this regard.

## 2. Research Aims and Questions

Numerous studies have explored the nature of CPS, some of them discussing and analysing it from behavioural or cognitive perspectives. However, there have barely been any that have merged these two perspectives. From the cognitive perspective, this study explores the role of thinking skills (including IR and CR) in the cognition process of CPS. From the behavioural perspective, the study focuses on students’ behaviour (i.e., their exploration strategy) in the CPS assessment process. More specifically, the research aims to fill this gap and examine students’ use of statistically distinguishable exploration strategies in CPS environments and to detect the connection between the level of students’ thinking skills and their behaviour strategies in the CPS environment. The following research questions were thus formed.

(RQ1) What exploration strategy profiles characterise the various problem-solvers at the university level?
(RQ2) Can developmental differences in CPS, IR and CR be detected among students with different exploration strategy profiles?
(RQ3) What are the similarities and differences in the roles IR and CR play in the CPS process as well as in the two phases of CPS (i.e., KAC and KAP) among students with different exploration strategy profiles?

## 3. Methods

### 3.1. Participants and Procedure

The sample was drawn from one of the largest universities in Hungary. Participation was voluntary, but students were able to earn one course credit for taking part in the assessment. The participants were students who had just started their studies there (N = 1671). 43.4% of the first-year students took part in the assessment. 50.9% of the participants were female, and 49.1% were male. We filtered the sample and excluded those who had more than 80% missing data on any of the tests. After the data were cleaned, data from 1343 students were available for analysis. The test was designed and delivered via the eDia online assessment system (Csapó and Molnár 2019). The assessment was held in the university ICT room and divided into two sessions. The first session involved the CPS test, whereas the second session entailed the IR and CR tests. Each session lasted 45 min. The language of the tests was Hungarian, the mother tongue of the students.

### 3.2. Instruments

#### 3.2.1. Complex Problem Solving (CPS)

The CPS assessment instrument adopted the MicroDYN approach. It contains a total of twelve scenarios, and each scenario consisted of two items (one item in the KAC phase and one item in the KAP phase in each problem scenario). Twelve KAC items and twelve KAP items were therefore delivered on the CPS test for a total of twenty-four items. Each scenario has a fictional cover story. For instance, students found a sick cat in front of their house, and they were expected to feed the cat with two different kinds of cat food to help it recover.

**Figure 1:**
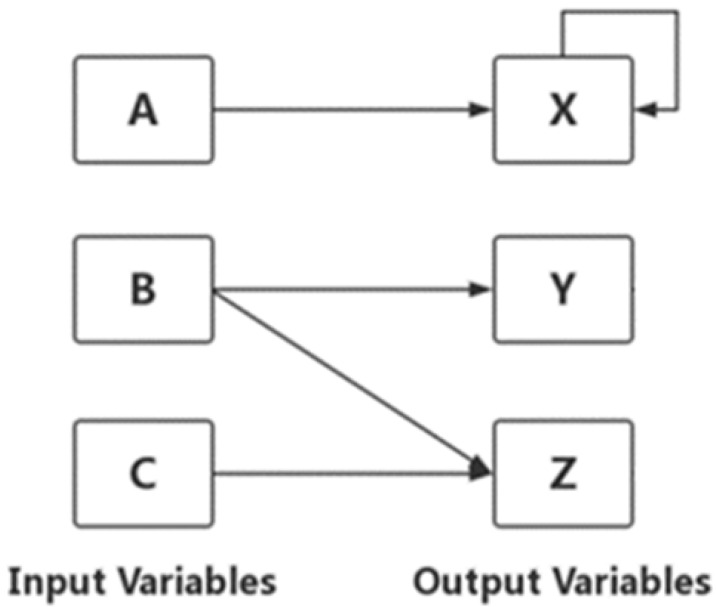
A typical MicroDYN structure with three input variables and three output variables (Greiff and Funke 2009).

Each item contains up to three input and three output variables. The relations between the input and output variables were formulated with linear structural equations (Funke 2001). Figure 1 shows a MicroDYN sample structure containing three input variables (A, B and C), three output variables (X, Y and Z) and a number of possible relations between the variables. The complexity of the item was defined by the number of input and output variables, and the number of relations between the variables. The test began with the item with the lowest complexity. The complexity of each item gradually increased as the test progressed.

**Figure 2:**
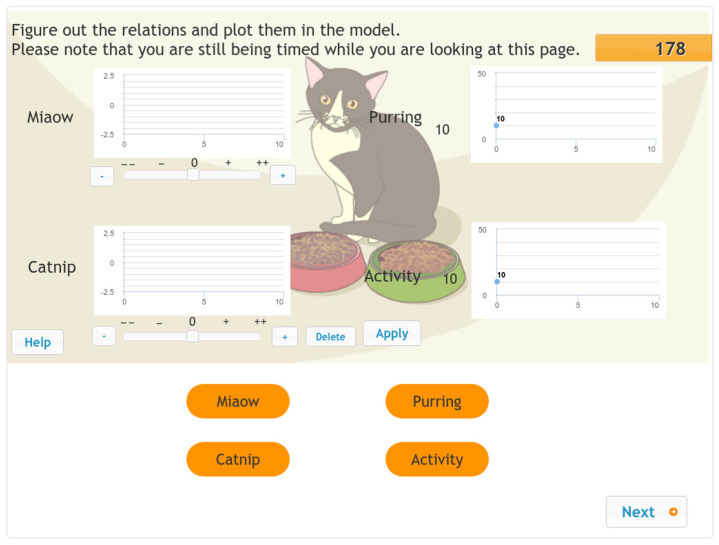
Screenshot of the MicroDYN item Cat—first phase (knowledge acquisition). (The items were administered in Hungarian.)

The interface of each item displays the value of each variable in both numerical and figural forms (See Figure 2). Each of the input variables has a controller, which makes it possible to vary and set the value between +2 (+ +) and −2 (− −). To operate the system, students need to click the “+” or “−” button or use the slider directly to select the value they want to be added to or subtracted from the current value of the input variable. After clicking the “Apply” button in the interface, the input variables will add or subtract the selected value, and the output variables will show the corresponding changes. The history of the values for the input and output variables within the same problem scenario is displayed on screen. If students want to withdraw all the changes and set all the variables to their original status, they can click the “Reset” button.

**Figure 3:**
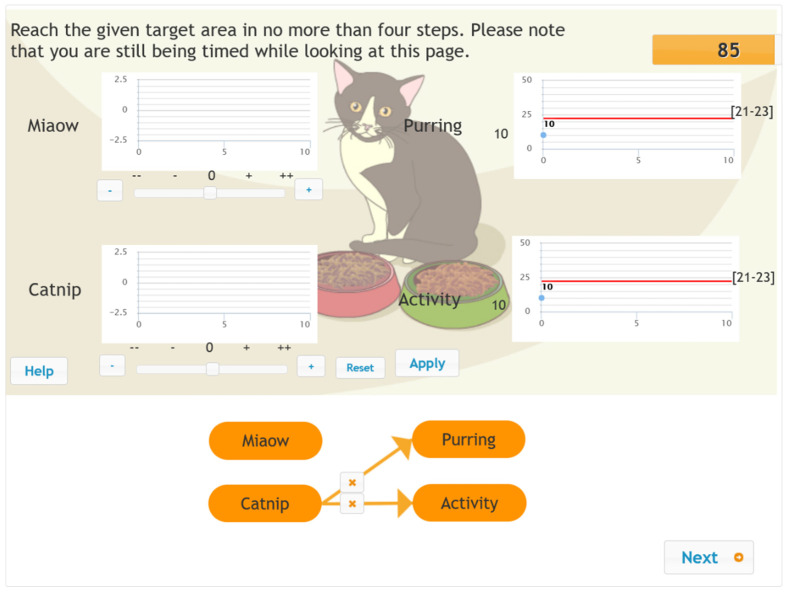
Screenshot of the MicroDYN item Cat—second phase (knowledge application). (The items were administered in Hungarian).

In the first phase of the problem-solving process, the KAC phase, students are asked to interact with the system by changing the value of the input variables and observing and analysing the corresponding changes in the output variables. They are then expected to determine the relationship between the input and output variables and draw it in the form of (an) arrow(s) on the concept map at the bottom of the interface. To avoid item dependence in the second phase of the problem-solving process, the students are provided with a concept map during the KAP phase (see Figure 3), which shows the correct connections between the input and output variables. The students are expected to interact with the system by manipulating the input variables to make the output variables reach the given target values in four steps or less. That is, they cannot click on the “Apply” button more than four times. The first phase had a 180 s time limit, whereas the second had a 90 s time limit.

#### 3.2.2. Inductive Reasoning (IR)

**Figure 4:**
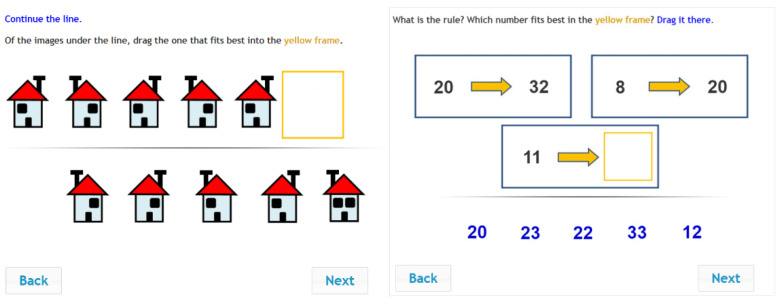
Sample items for the IR test. (The items were administered in Hungarian.).

The IR instrument (see Figure 4) was originally designed and developed in Hungary (Csapó 1997). In the last 25 years, the instrument has been further developed and scaled for a wide age range (Molnár and Csapó 2011). In addition, figural items have been added, and the assessment method has evolved from paper-and-pencil to computer-based (Pásztor 2016). Currently, the instrument is widely employed in a number of countries (see, e.g., Mousa and Molnár 2020; Pásztor et al. 2018; Wu et al. 2022; Wu and Molnár 2018). In the present study, four types of items were included after test adaptation: figural series, figural analogies, number analogies and number series. Students were expected to ascertain the correct relationship between the given figures and numbers and select a suitable figure or number as their answer. Students used the drag-and-drop operation to provide their answers. In total, 49 inductive reasoning items were delivered to the participating students.

#### 3.2.3. Combinatorial Reasoning (CR)

**Figure 5:**
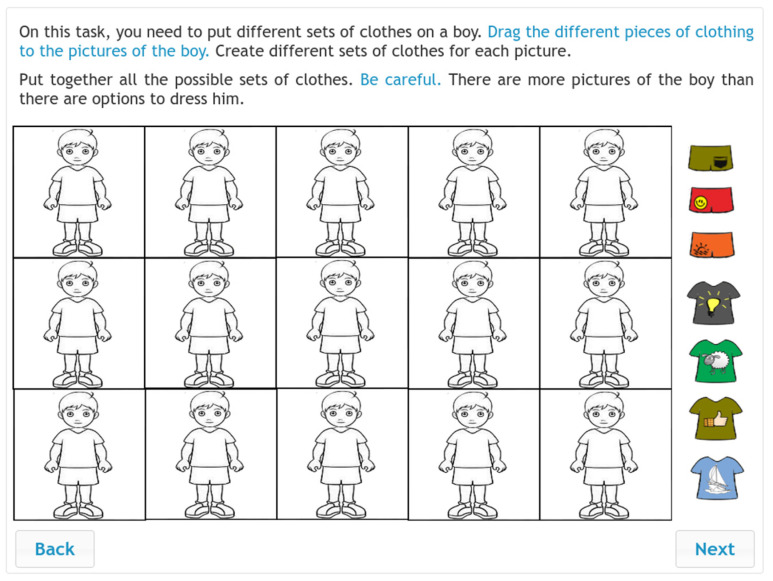
Sample item for the CR test. (The items were administered in Hungarian).

The CR instrument (see Figure 5) was originally designed by Csapó (1988). The instrument was first developed in paper-and-pencil format and then modified for computer use (Pásztor and Csapó 2014). Each item contained figural or verbal elements and a clear requirement for combing through the elements. Students were asked to list every single combination based on a given rule they could find. For the figural items, students provided their answers using the drag-and-drop operation; for the verbal items, they were asked to type their answers in a text box provided on screen. The test consisted of eight combinatorial reasoning items in total.

### 3.3. Scoring

Students’ performance was automatically scored via the eDia platform. Items on the CPS and IR tests were scored dichotomously. In the first phase (KAC) of the CPS test, if a student drew all the correct relations on the concept map provided on screen within the given timeframe, his/her performance was assigned a score of 1 or otherwise a score of 0. In the second phase (KAP) of the CPS test, if the student successfully reached the given target values of the output variables by manipulating the level of the input variables within no more than four steps and the given timeframe, then his/her performance earned a score of 1 or otherwise a score of 0. On the IR test items, if a student selected the correct figure or number as his/her answer, then he or she received a score of 1; otherwise, the score was 0.

Students’ performance on the CR test items was scored according to a special J index, which was developed by Csapó (1988). The J index ranges from 0 to 1, where 1 means that the student provided all the correct combinations without any redundant combinations on the task. The formula for computing the J index is the following:

J = x(T − y)/T^2^,

where

x stands for the number of correct combinations in the student’s answer,

T stands for the number of all possible correct combinations, and

y stands for the number of redundant combinations in the student’s answer. 

Furthermore, according to Csapó’s (1988) design, if y is higher than T, then the J index will be counted as 0.

### 3.4. Coding and Labelling the Logfile Data

Beyond concrete answer data, students’ interaction and manipulation behaviour were also logged in the assessment system. This made it possible to analyse students’ exploration behaviour in the first phase of the CPS process (KAC phase). Toward this aim, we adopted a labelling system developed by Molnár and Csapó (2018) to transfer the raw logfile data to structured data files for analysis. Based on the system, each trial (i.e., the sum of manipulations within the same problem scenario which was applied and tested by clicking the “Apply” button) was modelled as a single data entity. The sum of these trials within the same problem was defined as a strategy. In our study, we only consider the trials which were able to provide useful and new information for the problem-solvers, whereas the redundant or operations trials were excluded.

In this study, we analysed students’ trials to determine the extent to which they used the VOTAT strategy: fully, partially or not at all. This strategy is the most successful exploration strategy for such problems; it is the easiest to interpret and provides direct information about the given variable without any mediation effects (Fischer et al. 2012; Greiff et al. 2018; Molnár and Csapó 2018; Wüstenberg et al. 2014; Wu and Molnár 2021). Based on the definition of VOTAT noted in Section 1.3, we checked students’ trials to ascertain if they systematically varied one input variable while keeping the others unchanged, or applied a different, less successful strategy. We considered the following three types of trials:

1. “Only one single input variable was manipulated, whose relationship to the output variables was unknown (we considered a relationship unknown if its effect cannot be known from previous settings), while the other variables were set at a neutral value like zero […]
2. One single input variable was changed, whose relationship to the output variables was unknown. The others were not at zero, but at a setting used earlier. […]
3. One single input variable was changed, whose relationship to the output variables was unknown, and the others were not at zero; however, the effect of the other input variable(s) was known from earlier settings. Even so, this combination was not attempted earlier” (Molnár and Csapó 2018, p. 8)

We used the numbers 0, 1 and 2 to distinguish the level of students’ use of the most effective exploration strategy (i.e., VOTAT). If a student applied one or more of the above trials for every input variable within the same scenario, we considered that they had used the full VOTAT strategy and labelled this behaviour 2. If a student had only employed VOTAT on some but not all of the input variables, we concluded that they had used a partial VOTAT strategy for that problem scenario and labelled it 1. If a student had used none of the trials noted above in their problem exploration, then we determined that they had not used VOTAT at all and thus gave them a label of 0.

### 3.5. Data Analysis Plan

We used LCA (latent class analysis) to explore students’ exploration strategy profiles. LCA is a latent variable modelling approach that can be used to identify unmeasured (latent) classes of samples with similarly observed variables. LCA has been widely used in analysing logfile data for CPS assessment and in exploring students’ behaviour patterns (see, e.g., Gnaldi et al. 2020; Greiff et al. 2018; Molnár et al. 2022; Molnár and Csapó 2018; Mustafić et al. 2019; Wu and Molnár 2021). The scores for the use of VOTAT in the KAC phase (0, 1, 2; see Section 3.4) were used for the LCA analysis. We used Mplus (Muthén and Muthén 2010) to run the LCA analysis. Several indices were used to measure the model fit: AIC (Akaike information criterion), BIC (Bayesian information criterion) and aBIC (adjusted Bayesian information criterion). With these three indicators, lower values indicate a better model fit. Entropy (ranging from 0 to 1, with values close to 1 indicating high certainty in the classification). The Lo–Mendell–Rubin adjusted likelihood ratio was used to compare the model containing n latent classes with the model containing n − 1 latent classes, and the p value was the indicator for whether a significant difference could be detected (Lo et al. 2001). The results of the Lo–Mendell–Rubin adjusted likelihood ratio analysis were used to decide the correct number of latent classes in LCA models.

ANOVA was used to analyse the performance differences for CPS, IR and CR across the students from the different class profiles. The analysis was run using SPSS. A path analysis (PA) was employed in the structural equation modelling (SEM) framework to investigate the roles of CR and IR in CPS and the similarities and differences across the students from the different exploration strategy profiles. The PA models were carried out with Mplus. The Tucker–Lewis index (TLI), the comparative fit index (CFI) and the root-mean-square error of approximation (RMSEA) were used as indicators for the model fit. A TLI and CFI larger than 0.90 paired with a RMSEA less than 0.08 are commonly considered as an acceptable model fit (van de Schoot et al. 2012).

## 4. Results

### 4.1. Descriptive Results

All three tests showed good reliability (Cronbach’s α: CPS: 0.89; IR: 0.87; CR: 0.79). Furthermore, the two sub-dimensions of the CPS test, KAC and KAP, also showed satisfactory reliability (Cronbach’s α: KAC: 0.86; KAP: 0.78). The tests thus proved to be reliable. The means and standard deviations of students’ performance (in percentage) on each test are provided in Table 1.

### 4.2. Four Qualitatively Different Exploration Strategy Profiles Can Be Distinguished in CPS

Based on the labelled logfile data for CPS, we applied latent class analyses to identify the behaviour patterns of the students in the exploration phase of the problem-solving process. The model fits for the LCA analysis are listed in Table 2. Compared with the 2 or 3 latent class models, the 4 latent class model has a lower AIC, BIC and aBIC, and the likelihood ratio statistical test (the Lo–Mendell–Rubin adjusted likelihood ratio test) confirmed it has a significantly better model fit. The 5 and 6 latent class models did not show a better model fit than the 4 latent class model. Therefore, based on the results, four qualitatively different exploration strategy profiles can be distinguished, which covered 96% of the students.

**Figure 6:**
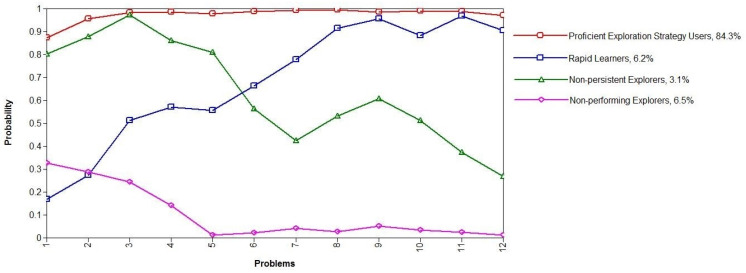
Four qualitatively different exploration strategy profiles.

The patterns for the four qualitatively different exploration strategy profiles are shown in Figure 6. In total, 84.3% of the students were proficient exploration strategy users, who were able to use VOTAT in each problem scenario independent of its difficulty level (represented by the red line in Figure 5). In total, 6.2% of the students were rapid learners. They were not able to apply VOTAT at the beginning of the test on the easiest problems but managed to learn quickly, and, after a rapid learning curve by the end of the test, they reached the level of proficient exploration strategy users, even though the problems became much more complex (represented by the blue line). In total, 3.1% of the students proved to be non-persistent explorers, and they employed VOTAT on the easiest problems but did not transfer this knowledge to the more complex problems. Finally, they were no longer able to apply VOTAT when the complexity of the problems increased (represented by the green line). In total, 6.5% of the students were non-performing explorers; they barely used any VOTAT strategy during the whole test (represented by the pink line) independent of problem complexity.

### 4.3. Better Exploration Strategy Users Showed Better Performance in Reasoning Skills

Students with different exploration strategy profiles showed different kinds of performance in each reasoning skill under investigation. Results (see Table 3) showed that more proficient strategy users tended to have higher achievement in all the domains assessed as well as in the two sub-dimensions in CPS (i.e., KAC and KAP; ANOVA: CPS: F(3, 1339) = 187.28, *p* < 0.001; KAC: F(3, 1339) = 237.15, *p* < 0.001; KAP: F(3, 1339) = 74.91, *p* < 0.001; IR: F(3, 1339) = 48.10, *p* < 0.001; CR: F(3, 1339) = 28.72, *p* < 0.001); specifically, students identified as “proficient exploration strategy users” achieved the highest level on the reasoning skills tests independent of the domains. On average, they were followed by rapid learners, non-persistent explorers and, finally, non-performing explorers. Tukey’s post hoc tests revealed more details on the performance differences of students with different exploration profiles in each of the domains being measured. Proficient strategy users proved to be significantly more skilled in each of the reasoning domains. They were followed by rapid learners, who outperformed non-persistent explorers and non-performing explorers in CPS. In the domains of IR and CR, there were no achievement differences between rapid learners and non-persistent explorers, who significantly outperformed non-performing strategy explorers.

### 4.4. The Roles of IR and CR in CPS and Its Processes Were Different for Each Type of Exploration Strategy User

**Figure 7:**
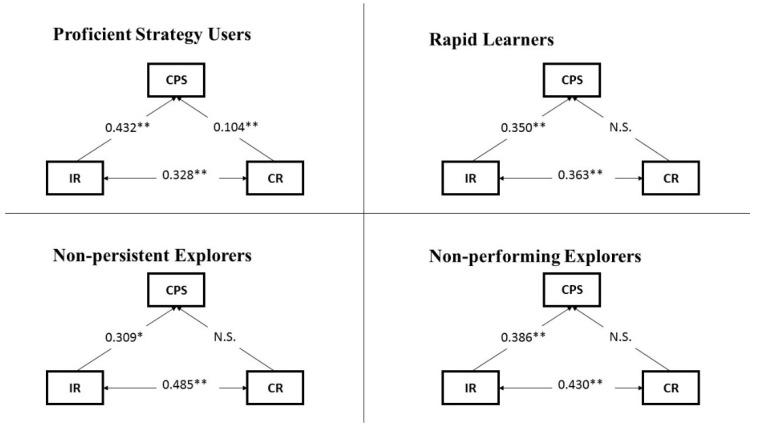
Path analysis models (with CPS, IR and CR) for each type of strategy user; * significant at 0.05 (*p*  <  0.05); ** significant at 0.01 (*p*  <  0.01); N.S.: no significant effect can be found.

**Figure 8:**
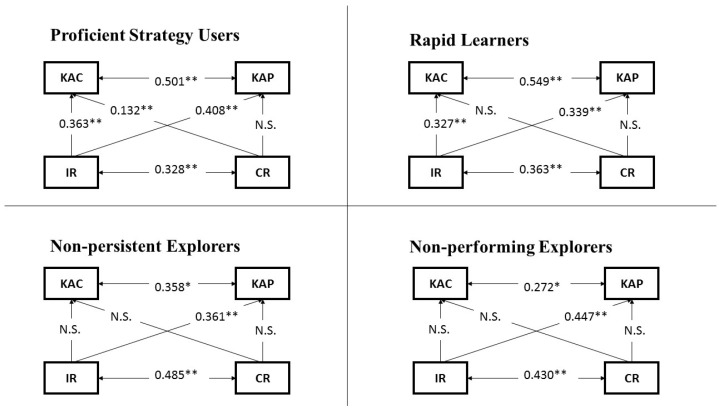
Path analysis models (with KAC, KAP, IR and CR) for each type of strategy user; * significant at 0.05 (*p*  <  0.05); ** significant at 0.01 (*p*  <  0.01); N.S.: no significant effect can be found.

Path analysis was used to explore the predictive power of IR and CR for CPS and its processes, knowledge acquisition and knowledge application, for each group of students with different exploration strategy profiles. That is, four path analysis models were built to indicate the predictive power of IR and CR for CPS (see Figure 7), and another four path analyses models were developed to monitor the predictive power of IR and CR for the two empirically distinguishable phases of CPS (i.e., KAC and KAP) (see Figure 8). All eight models had good model fits, the fit indices TLI and CFI were above 0.90, and RMSEA was less than 0.08.

Students’ level of IR significantly predicted their level of CPS in all four path analysis models independent of their exploration strategy profile (Figure 7; proficient strategy users: β = 0.432, *p* < 0.01; rapid learners: β = 0.350, *p* < 0.01; non-persistent explorers: β = 0.309, *p* < 0.05; and non-performing explorers: β = 0.386, *p* < 0.01). This was not the case for CR, which only proved to have predictive power for CPS among proficient strategy users (β = 0.104, *p* < 0.01). IR and CR were significantly correlated in all four models.

After examining the roles of IR and CR in the CPS process, we went further to explore the roles of these two reasoning skills in the distinguishable phases of CPS. The path analysis models (Figure 8) showed that the predictive power of IR and CR for KAC and KAP was varied in each group. Levels of IR and CR among non-persistent explorers and non-performing explorers failed to predict their achievement in the KAC phase of the CPS process. Moreover, rapid learners’ level of IR significantly predicted their achievement in the KAC phase (β = 0.327, *p* < 0.01), but their level of CR did not have the same predictive power. Furthermore, the proficient strategy users’ levels of both reasoning skills had significant predictive power for KAC (IR: β = 0.363, *p* < 0.01; CR: β = 0.132, *p* < 0.01). In addition, in the KAP phase of the CPS problems, IR played a significant role for all types of strategy users, although with different power (proficient strategy users: β = 0.408, *p* < 0.01; rapid learners: β = 0.339, *p* < 0.01; non-persistent explorers: β = 0.361, *p* < 0.01; and non-performing explorers: β = 0.447, *p* < 0.01); by contrast, CR did not have significant predictive power for the KAP phase in any of the models.

## 5. Discussion

The study aims to investigate the role of IR and CR in CPS and its phases among students using statistically distinguishable exploration strategies in different CPS environments. We examined 1343 Hungarian university students and assessed their CPS, IR and CR skills. Both achievement data and logfile data were used in the analysis. The traditional achievement indicators formed the foundation for analysing the students’ CPS, CR and IR performance, whereas process data extracted from logfile data were used to explore students’ exploration behaviour in various CPS environments.

Four qualitatively different exploration strategy profiles were distinguished: proficient strategy users, rapid learners, non-persistent explorers and non-performing explorers (RQ1). The four profiles were consistent with the result of another study conducted at university level (see Molnár et al. 2022), and the frequencies of these four profiles in these two studies were very similar. The two studies therefore corroborate and validate each other’s results. The majority of the participants were identified as proficient strategy users. More than 80% of the university students were able to employ effective exploration strategies in various CPS environments. Of the remaining students, some performed poorly in exploration strategy use in the early part of the test (rapid learners), some in the last part (non-persistent explorers) and some throughout the test (non-performing explorers). However, students with these three exploration strategy profiles only constituted small portions of the total sample (with proportions ranging from 3.1% to 6.5%). The university students therefore exhibited generally good performance in terms of exploration strategy use in a CPS environment, especially compared with previous results among younger students (e.g., primary school students, see Greiff et al. 2018; Wu and Molnár 2021; primary to secondary students, see Molnár and Csapó 2018).

The results have indicated that better exploration strategy users achieved higher CPS performance and had better development levels of IR and CR (RQ2). First, the results have confirmed the importance of VOTAT in a CPS environment. This finding is consistent with previous studies (e.g., Greiff et al. 2015a; Molnár and Csapó 2018; Mustafić et al. 2019; Wu and Molnár 2021). Second, the results have confirmed that effective use of VOTAT is strongly tied to the level of IR and CR development. Reasoning forms an important component of human intelligence, and the level of development in reasoning was an indicator of the level of intelligence (Klauer et al. 2002; Sternberg and Kaufman 2011). Therefore, this finding has supplemented empirical evidence for the argument that effective use of VOTAT is associated with levels of intelligence to a certain extent.

The roles of IR and CR proved to be varied for each type of exploration strategy user (RQ3). For instance, the level of CPS among the best exploration strategy users (i.e., the proficient strategy users) was predicted by both the levels of IR and CR, but this was not the case for students with other profiles. In addition, the results have indicated that IR played important roles in both the KAC and KAP phases for the students with relatively good exploration strategy profiles (i.e., proficient strategy users and rapid learners) but only in the KAP phase for the rest of the students (non-persistent explorers and non-performing explorers); moreover, the predictive power of CR can only be detected in the KAC phase of the proficient strategy users. To sum up, the results suggest a general trend of IR and CR playing more important roles in the CPS process among better exploration strategy users.

Combining the answers to RQ2 and RQ3, we can gain further insights into students’ exploration strategy use in a CPS environment. Our results have confirmed that the use of VOTAT is associated with the level of IR and CR development and that the importance of IR and CR increases with proficiency in exploration strategy use. Based on these findings, we can make a reasonable argument that IR and CR are essential skills for using VOTAT and that underdeveloped IR and CR will prevent students from using effective strategies in a CPS environment. Therefore, if we want to encourage students to become better exploration strategy users, it is important to first enhance their IR and CR skills. Previous studies have suggested that establishing explicit training in using effective strategies in a CPS environment is important for students’ CPS development (Molnár et al. 2022). Our findings have identified the importance of IR and CR in exploration strategy use, which has important implications for designing training programmes.

The results have also provided a basis for further studies. Future studies have been suggested to further link the behavioural and cognitive perspectives in CPS research. For instance, IR and CR were considered as component skills of CPS (see Section 1.2). The results of the study have indicated the possibility of not only discussing the roles of IR and CR in the cognitive process of CPS, but also exploration behaviour in a CPS environment. The results have thus provided a new perspective for exploring the component skills of CPS.

## 6. Limitations

There are some limitations in the study. All the tests were low stake; therefore, students might not be sufficiently motivated to do their best. This feature might have produced the missing values detected in the sample. In addition, some students’ exploration behaviour shown in this study might theoretically be below their true level. However, considering that data cleaning was adopted in this study (see Section 3.1), we believe this phenomenon will not have a remarkable influence on the results. Moreover, the CPS test in this study was based on the MicroDYN approach, which is a well-established and widely used artificial model with a limited number of variables and relations. However, it does not have the power to cover all kinds of complex and dynamic problems in real life. For instance, the MicroDYN approach cannot measure ill-defined problem solving. Thus, this study can only demonstrate the influence of IR and CR on problem solving in well-defined MicroDYN-simulated problems. Furthermore, VOTAT is helpful with minimally complex problems under well-defined laboratory conditions, but it may not be that helpful with real-world, ill-defined complex problems (Dörner and Funke 2017; Funke 2021). Therefore, the generalizability of the findings is limited.

## 7. Conclusions

In general, the results have shed new light on students’ problem-solving behaviours in respect of exploration strategy in a CPS environment and explored differences in terms of the use of thinking skills between students with different exploration strategies. Most studies discuss students’ problem-solving strategies from a behavioural perspective. By contrast, this paper discusses them from both behavioural and cognitive perspectives, thus expanding our understanding in this area. As for educational implications, the study contributes to designing and revising training methods for CPS by identifying the importance of IR and CR in exploration behaviour in a CPS environment. To sum up, the study has investigated the nature of CPS from a fresh angle and provided a sound basis for future studies.

## Figures and Tables

**Table 1:** The means and standard deviations of students’ performance on each test.

	CPS			IR	CR
	Overall	KAC	KAP		
Mean (%)	56.21	62.93	49.50	65.83	68.46
S.D. (%)	22.37	26.65	22.75	15.41	20.02

**Table 2:** Fit indices for latent class analyses.

Number of Latent Classes	AIC	BIC	aBIC	Entropy	L–M–R Test	*p*
2	9078	9333	9177	0.987	4255	<0.001
3	8520	8905	8670	0.939	604	<0.001
4	8381	8897	8582	0.959	188	<0.05
5	8339	8984	8591	0.955	92	0.93
6	8309	9084	8611	0.877	96	0.34

**Table 3:** Students’ performance on each test—grouped according to the different exploration strategy profiles.

Class Profiles		CPS			IR	CR
		Overall	KAC	KAP		
Proficient strategy users	Mean (%)	61.37	69.57	53.17	67.79	70.47
	S.D. (%)	19.67	22.25	21.90	14.22	18.96
Rapid learners	Mean (%)	35.39	36.65	34.14	59.23	62.67
	S.D. (%)	14.26	20.45	17.15	14.22	17.60
Non-persistent explorers	Mean (%)	27.03	24.59	29.47	57.29	56.11
	S.D. (%)	10.75	14.06	11.80	18.75	24.52
Non-performing explorers	Mean (%)	22.75	19.64	25.86	50.65	53.72
	S.D. (%)	12.67	15.30	16.38	16.55	23.99

## Data Availability

The data used to support the findings cannot be shared at this time as it also forms part of an ongoing study.
